# School and Healthcare Collaboration in Implementing and Sustaining School‐Based Health Centers in Rural Communities

**DOI:** 10.1111/josh.70085

**Published:** 2025-09-01

**Authors:** Xue Zhang, Mildred E. Warner, Chen Wu

**Affiliations:** ^1^ The Pennsylvania State University University Park Pennsylvania USA; ^2^ Cornell University Ithaca New York USA

**Keywords:** collective impact, communication, confidentiality, rural health, school‐based health centers, trust

## Abstract

**Background:**

This study examines how school‐based health centers (SBHCs) are implemented and sustained through schools and a healthcare system in a four‐county rural region in New York State.

**Methods:**

Twelve semi‐structured interviews were conducted with school superintendents and employees of the healthcare system, including SBHC medical providers (physician assistant, mental health therapist, nurse), SBHC manager, care coordinator, Chief of Pediatrics, and executive leader between January and April 2024. Interviews were thematically coded using collective impact and collective action frameworks.

**Results:**

SBHCs are implemented and sustained through a strong collaborative culture, shared financial, physical, and human resources, and a common agenda for student well‐being. Trust and open communication were essential in clarifying roles and overcoming institutional challenges. Key barriers included funding for startup costs, challenges in data sharing and confidentiality, and different institutional priorities. Regular communication, local governmental support, and community trust helped mitigate these challenges.

**Implications for School Health Policy, Practice, and Equity:**

Policies should support schools and healthcare systems as equal partners, ensure sustainable funding, and provide clearer guidance on data sharing to advance health equity in rural areas.

**Conclusions:**

Collaborative partnerships, trust, and open communication between schools and healthcare systems are essential to support SBHCs in rural communities.

## 1 Introduction

School‐based health centers (SBHCs) are unique healthcare settings implemented and sustained through school‐healthcare collaboration to facilitate access for underserved children and youth [1, 2]. Typically located in school buildings, SBHCs are healthcare facilities that use a specific collaborative model of care to address access barriers and provide comprehensive services, including primary, behavioral, and dental care [1, 3, 4]. SBHCs, combined with family and youth engagement and care coordination, play an important role in addressing health disparities in underserved rural communities facing adverse social determinants of health, where there is a lack of healthcare access, infrastructure, and social services [5, 6].

Although a growing number of studies have examined the impact of SBHCs on healthcare utilization and educational outcomes [2, 4, 7, 8, 9, 10], few have focused on how SBHCs are implemented and sustained. The number of SBHCs was more than tripled from 1135 in 1998–99 to about 3900 in 2022 [2, 7]. However, data from the National Center for Education Statistics reported 90,604 schools operating in the 2022–23 school year [11], indicating only 4% of schools had an SBHC. Funding is a key challenge of both opening and sustaining SBHCs. A review conducted in 2014 shows that the startup cost can be $378,704 per SBHC, and the operation cost can reach $659,684 per SBHC per year [12]. In addition, most SBHCs sustain their operations primarily through insurance billings [2], but many SBHC users are uninsured, underinsured, or from low‐income families [3, 7, 8]. Other key factors in implementing SBHCs include securing strong partnerships between schools and healthcare systems, engaging in public relationships and health education, conducting extensive planning, and gaining community buy‐in and support [13, 14, 15]. In contrast, limited time and resources are major barriers to opening SBHCs [3]. After implementation, SBHCs may continue to face additional challenges to sustain operations, including lack of understanding about the differences between SBHCs and other school‐health resources, lack of communication between schools and healthcare systems, differing priorities of health and education systems, issues of confidentiality and data sharing, and building trust with parents and community [3, 8, 15]. For example, Johnson et al. [15] evaluated the implementation and sustainability of SBHCs in urban, semi‐urban, and rural communities, and found rural SBHCs struggle to enroll students due to trust issues, as communities are hesitant to use SBHC services staffed by outsiders. Operating and sustaining SBHCs requires ongoing government funding, support from policymakers, continued financial contributions from donors, and buy‐in from schools, community organizations, and families [4, 13].

To comprehensively examine factors that contribute to successful school‐healthcare collaboration in implementing and sustaining SBHCs in rural communities, this study integrates collective action theory with the collective impact framework, and conducts a case study in a four‐county rural region in New York State. In 2023, New York State had 247 approved SBHCs operating, with 19% located in rural areas [16]. To implement an SBHC, schools need to partner with providers defined in Article 28 of the Public Health Law in New York State, including accredited hospitals, treatment facilities, or clinics such as Federally Qualified Health Centers [17, 18]. The collective action theory and collective impact framework together guide our assessment of SBHCs implementation and sustainability. Collective action theory is a governance framework that explains how cooperation among diverse stakeholders is achieved through reputation, trust and reciprocity [19]. It provides insights into the internal dynamics of stakeholder relationships, illustrating how reputation builds trust which leads to reciprocity, and reciprocity results in successful collaboration [19]. This internal focus ensures that the foundational elements of collaboration are strong and sustainable. The collective impact framework functions as a systematic approach to achieving large‐scale social change through multi‐sector collaboration [20]. It addresses the broader, systemic aspects of collaboration through a common agenda, shared measurement, mutually reinforcing activities, ongoing communication, and an independent backbone organization [20].

Collective action and collective impact framework address different aspects of collaboration. For example, trust is a critical component in maintaining strong, ongoing collaboration in the collective action framework [19]. However, elements such as a common agenda and shared measurement are only discussed in collective impact theory [20]. By integrating both frameworks, we analyze the interplay between internal governance and external coordination, leading to a more holistic understanding of school‐healthcare partnerships in rural settings. This case study provides an opportunity to evaluate both frameworks in the context of implementing and sustaining SBHCs.

## 2 Methods

### 2.1 Study Area

We conducted semi‐structured interviews to explore how schools and a healthcare network collaborated to implement and sustain SBHCs in a four‐county rural region in New York State. The Bassett Healthcare Network, defined as a healthcare collaborator in NYS Article 28, serves as the main healthcare system in the region. As of 2023, Bassett operated 21 SBHCs across 17 of 38 school districts in these counties, with the first SBHC established in 1992. In 2023, 81% of students in these 17 school districts were enrolled in the SBHC.

### 2.2 Instrumentation and Procedure

Data were collected from 12 semi‐structured interviews, involving five school superintendents from four schools with SBHCs and one school planning to open an SBHC; three SBHC medical providers (a physician assistant, a mental health therapist, and a licensed practical nurse); three SBHC leaders (a SBHC manager, the Chief of Pediatrics, and a senior vice president at Bassett), and one care coordinator. Participants were identified using snowball sampling, starting with a pilot interview with the SBHC manager, who then recommended additional interviewees. In each county, we interviewed at least one school superintendent and at least one SBHC medical provider. Although our sample size is relatively small, our research focuses on a specific school‐healthcare collaboration model and includes shareholders directly involved in SBHC implementation and operation. Guided by collective action theory and the collective impact framework, thematic convergence (e.g., shared resources, trust, communication) was observed across interviewees from both schools and the healthcare system. Prior research also suggests that thematic saturation can be reached with 12 interviews [21, 22].

This study was exempted by the Cornell Institutional Review Board. Consent forms were sent to interviewees before the interviews, and oral consent was obtained before recording. The interviews were conducted between January 2024 and April 2024, each lasting 60 min. We asked interviewees about their motivations for collaboration, the challenges they encountered, and their vision for improving collaboration and sustaining the SBHC over time. Specifically, we asked:

1. Why does your school/Bassett support the SBHC?
2. What are the costs and benefits of having a SBHC?
3. What do you see as important factors or challenges in school‐healthcare collaboration?
4. What challenges do you face? What helps address these challenges?
5. If the collaboration were to be different than it is now, what would you like to see changed?

### 2.3 Data Analysis

Each interview was recorded via Zoom, transcribed, and discussed immediately afterward. A coding structure was developed based on the frameworks of collective impact, including predefined categories: common agenda, shared measures, reinforcing activities, communication, and backbone support, then linked to the core relationships from collective action theory, including reputation, trust, and reciprocity. The transcripts were open‐coded, with coding labels assigned to individual sentences. These codes were then grouped into each predefined category, and thematic coding was performed within each category. The list of codes and themes was then discussed, and disagreements were resolved within the research group. Finally, a lead researcher employed qualitative software (NVivo 14) to deductively code the interviews, resulting in a final set of themes.

## 3 Results

### 3.1 Backbone Support: Shared Resources and Collaborative Culture

Interviews show that SBHCs are built and sustained through a collaborative culture and shared resources between two backbone organizations: schools and the Bassett Healthcare Network. School superintendents and the SBHC manager noted that to build an SBHC, schools provide office space, lump‐sum start‐up funds for space renovation, a yearly flat fee, and other services to support a primary care setting, including cleaning, electricity, and safety. Bassett is responsible for all medical resources, including doctors, nurses, IT and data systems, and equipment, and covers out‐of‐pocket costs by using a combination of insurance billing, grant funding through New York State, and the annual flat fee from schools.

Interviews with school superintendents show that although Bassett's requirements, such as start‐up fees and office space, are essential to provide health care services, they can create a great financial burden for rural schools. A school superintendent seeking to establish a SBHC mentioned the start‐up cost challenges ($1 million to set up the office and an additional $600,000 to fund the first 3 years).

A common theme mentioned by interviewees is the strong collaborative culture between schools, the healthcare system, and government officials, which helps overcome financial constraints. A school superintendent noted they worked on building a SBHC for almost a decade, but were lacking the start‐up cost ($300,000 at that time). They worked with local legislators to advocate for support for the high needs of the district due to lack of healthcare access and transportation, and working parents struggling to get their children to appointments. The state senator for the region spent two years putting money aside to support the startup fee. The school was able to cut back on administration by combining an elementary principal and a middle‐high principal, and worked with Bassett to transform the extra office space to be exactly what they needed. The SBHC manager mentioned that Bassett collaborates with schools to get funding for building SBHCs, for example, through the Health Resource and Service Administration (HRSA). Schools cannot apply for HRSA because they are not a healthcare organization. But Bassett can apply for HRSA grants and help schools build SBHCs.

### 3.2 Common Agenda and Mutually Reinforcing Activities: Reciprocity and Conflicts

#### 3.2.1 Reciprocity

Interviews with school superintendents and SBHC leaders show schools and Bassett have different missions, but share a common goal: to do what is best for students. This common goal is supported by a collaborative network that facilitates mutually reinforcing activities, involving SBHC leaders, care providers, school nurses, teachers, and superintendents. For example, a SBHC medical provider noted “we have incredible collaboration with school nurses…No one in the school knows every student in every family better than a school nurse…It [collaboration] also is very good with the school counseling department. They also are always looking for partners.” A SBHC medical provider also noted that the SBHC model allows them as clinicians to have more fidelity in the way they practice healthcare, as the school helps ensure follow‐through with the children.

Interviews with school superintendents show that schools benefit from the SBHCs in accessing physical, mental, and dental care, and improving attendance. Superintendents stated that “SBHC offers the flexibility to call the student down… every student is within 2 min of the clinic.”, and mentioned that SBHCs enable students to reduce out‐of‐class time for medical appointments. SBHC medical providers also noted that SBHCs provide regular teeth cleanings and mental health counseling for students. There are no out‐of‐pocket costs for visiting the SBHC. SBHCs take insurance, but other costs, such as co‐pays and balanced billing, do not get charged to the families, which is essential for children from low‐income families in rural communities to access care.

Interviews show that Bassett's investment in SBHCs is motivated by its mission for social benefits and a better strategic relationship with the public. A senior vice president at Bassett stated “One is, fundamentally, it is part of our mission. We do know that we help keep kids out of the emergency room, and we provide care that no one else could provide.” SBHCs also help Bassett's relationship with government leaders and future clients. A superintendent stated that, “The Board of Education sees the value.” A SBHC medical provider also noted “For the Bassett side, they love to publicize all the good things that we do. You know, I think that we generate a positive impression of Bassett from the time the child is 5 years old until they graduate. We really kind of have wrap‐around care so it can funnel into the larger healthcare organization.”

#### 3.2.2 Conflicts

Interviewees mentioned that different priorities and policies can create conflicts between healthcare providers and schools. A SBHC mental health provider noted that although the schools' goal is to keep students in class, it is difficult [for SBHC staff] to send students back to school after handling a fairly traumatic event. Also, SBHC staff are governed by a different compliance system than that of the schools. Healthcare professionals handle mandated reporting differently from the school (Health Insurance Portability and Accountability Act (HIPAA) versus Family Educational Rights and Privacy Act (FERPA) rules). So sometimes extra communication is needed to help the school understand the differences in best healthcare practice.

Interviewees also stated that it can be challenging to recognize that the schools and the SBHCs are different organizations. For example, a SBHC medical provider mentioned that they are employees of Bassett, not employees of school, but they are sometime mistaken for a “super school nurse.” They do not replace the school nurses, but instead work together to support students' health. A SBHC care coordinator noted that communication has improved over time: “At first and still continuing with some schools, there was a confusion between the role of the school nurse and school‐based health… The communication between those two groups has really improved.”

### 3.3 Shared Measurement: Building Trust and Addressing Confidentiality Concerns

#### 3.3.1 Shared Measurement: Enrollment Rate and Challenges

School superintendents mentioned that Bassett targets a 75% enrollment rate within the first 3 years, and building trust between SBHCs and students' parents and guardians is important to reach the enrollment rate. A school superintendent emphasized the importance of trust “… trust that we [school] already had established with the community. Then the school was able to kind of convince people to enroll their children…So I know our district has hit this well over the 75% faster than 3 years.” Superintendents also mentioned that they engaged the SBHC staff during school open houses and gave applications for SBHC enrollment as part of the school enrollment package. The school and SBHC also hold formal regional meetings twice a year to discuss strategies to improve the enrollment rate.

Interviews show that other measures, such as the improvement in students' health and attendance, are more difficult to quantify. Bassett's Chief of Pediatrics noted “Real dollars and the perception of efficiency with the care that they [SBHCs] provide is a cost that Bassett weighs in against the benefit of the preventive care that's being done and the service to the community…[preventive care is] almost obvious, just difficult to quantify.”

#### 3.3.2 Balancing Shared Measures and Confidentiality

We found there is a clear boundary in data sharing between schools and SBHCs to ensure students' confidentiality. The SBHC manager noted “I think that's [confidentiality] reassuring to our patients because they know what is told to us stays with us. It's never going to go any further unless those magic two exceptions, if they're going to hurt themselves or others, then all rules go out the door, and we make that clear right at the very beginning.” However, confidentiality is not a barrier to communication. A school superintendent noted that the school worked closely with the Bassett team to talk about students. While respecting privacy and legal requirements (HIPAA and FERPA), the conversations ensure that both the school and the SBHC respect confidentiality while meeting the needs of students.

### 3.4 Continuous Communication

Interviews show that communication supports all aspects of the SBHC‐healthcare collaboration. A school superintendent noted that the key to a successful partnership is open communication, “we found that regular meetings are important for communication.” A SBHC medical provider also noted “One thing that I find really helps is, at my site, we meet with school administration and the guidance team every other week for a meeting. We go over cases that the school [is] working with students of concern. That allows me to get updates from the school in a way where we have a set aside time to collaborate. So that's a piece where I really feel like [communication] is super helpful.”

There are a few barriers to communication identified by the SBHC manager, care coordinator, and medical providers: (1) staff turnover and position changes within the organization; (2) hybrid work leads to increased reliance on email, which may not receive immediate responses; (3) remote working staff may lack local knowledge; (4) it is difficult to schedule meetings. The SBHC manager noted that community collaboration during the pandemic was excellent, when there was a great need. However, after the pandemic, people do not have much patience for meetings.

## 4 Discussion

We examined the school‐healthcare collaboration in implementing and sustaining SBHCs using the collective action and collective impact framework. We found that the school‐healthcare collaboration happened within a complex system, built on bilateral shared resources between two backbone organizations rather than a single backbone organization (Table 1). The involvement of multiple backbone organizations requires a power balance between autonomy and interdependence [23]. We found that open communication, regular meetings, and clear boundaries help balance autonomy and interdependence. School‐healthcare collaboration functions through interconnected components of reciprocity, common agenda, shared measures, mutually reinforcing activities, and continuous communication (Table 1). These interconnections are powerful. They align with the collective action framework, where core and external relationships operate as an integrated system rather than isolated units [19]. The connections between these key dimensions are key to supporting the collaborative relationship.

**TABLE 1:** School‐healthcare collaboration.

	Applicability to school‐healthcare collaboration for SBHCs
Collective impact framework	
Single backbone organization	Two backbone organizations, shared resources
Common vision and mutually reinforcing activities	Shared goal but different missions
Continuous communication	Open communication, regular meetings, and clear boundaries
Shared measurement	Enrollment rate—easy to measure Economic and social benefits—harder to quantify Different policy rules (HIPPA and FERPA)
Collective action framework	
Trust	Critical between schools and healthcare collaborators, but also parents and community
Reciprocity	Critical for both resources and communication
Reputation	Critical for maintaining the partnership over time

*Source:* Authors analysis.

Shared measures in the school‐healthcare collaboration are challenging (Table 1). Schools and healthcare collaborators have different missions and priorities, different and sometimes incompatible reporting systems, and different concerns over privacy and confidentiality [24]. For example, challenges arise from balancing HIPAA compliance with the school's interest in addressing the needs of students. The Federal government could help schools and SBHCs with updated guidance on information sharing without compromising privacy [24]. It is also difficult to measure the economic and social benefits of SBHCs, as found in other studies [12]. Other social benefits, such as a decrease in absenteeism and an increase in educational outcomes, need to be further examined. Another challenge is the lack of standard measures of school‐healthcare collaboration. The student enrollment rate is the clear common measure in our study site. Other measures could be added to evaluate the effectiveness of SBHCs, such as the well‐child visits and the annual risk assessment from National Performance Measures (NPM) for SBHCs [25, 26].

The culture of collaboration, embedded in strong interpersonal ties and trust, is a key community asset to help rural communities collaborate across organizational boundaries. We found that trust and familiarity among residents foster school‐healthcare collaboration (Table 1). Research shows that community buy‐in and support are essential to build and sustain SBHCs [15]. It is also important to reach out to political leaders [17, 27]. As shown in our interviews, the school district, local, and state governments helped with the start‐up fee. This suggests that achieving public health goals relies on collaboration between public health and governmental systems, as the public policy decisions, including the allocation of scarce public resources, are made by public officials [27].

School‐healthcare collaboration needs a more community‐centered and policy‐informed approach. Our interviews show that the top‐down structure of the collective impact framework often overlooks the interests of diverse community members [28, 29], while parents and community members' support is essential to sustain the SBHC operation. The time and resources required by the collective impact framework may also limit the ability of small and under‐resourced communities to participate [29, 30], such as the rural communities in our study. Both the collective impact and collective action frameworks emphasize local collaboration but give less attention to broader policy change [31, 32]. State‐level policies could have a great impact on the implementation and sustainability of SBHC, such as Medicaid expansion, CHIP eligibility, SBHC policies on parental consent [33], and SBHC services such as family planning counseling and substance abuse counseling [34]. While collective impact provides a foundation for collaboration, such collaboration is fragmented and overly hierarchical [29, 31], suggesting a need for a more integrated, policy‐oriented approach to understanding and supporting SBHC‐healthcare collaboration.

SBHCs are important safety nets for underserved children in rural communities. Many families and students from economically disadvantaged groups live in our study area [5]. These groups are particularly vulnerable and are adversely affected by barriers to accessing healthcare services. As noted by our interviewees, SBHCs can be their only source of care. Addressing these disparities requires an approach that goes beyond standard collaborative models and integrates social justice to ensure that collaboration not only promotes shared goals but actively reduces disparities in healthcare access and improves health outcomes for underserved communities. SBHCs and schools do their part, but broader policy change is needed. Neither collective impact theory nor collective action theory directly addresses concerns of social justice in the process and outcomes of collaboration [29, 32]. An explicit social justice and equity lens is needed to address health disparities [35], and to support SBHCs.

## 5 Implications for School Health Policy, Practice, and Equity

Our study highlights several implications for school health policy and practice, particularly in rural communities where resources are limited. We found that successful implementation and sustainability of SBHCs requires a collaborative network that goes beyond a single backbone organization (e.g., school or healthcare system). Policies that aim to build and expand SBHCs should recognize schools and health systems as equal partners in a dynamic and reciprocal relationship, and support the sharing of financial, physical, and human resources.

Actively involving local government and community stakeholders is important for rural communities to overcome constraints. Our study shows that local school leaders, working in partnership with political representatives, successfully secured funding and adapted school infrastructure to meet healthcare needs. This emphasizes the importance of embedding SBHC policy within broader local government and public health frameworks.

Shared measurement remains a persistent challenge. We found that confidentiality and differing priorities between schools and healthcare systems can make it difficult to assess the effectiveness of SBHCs. To strengthen accountability and transparency, state and federal agencies should provide clearer guidance on data sharing that complies with HIPAA, and invest in systems that facilitate secure information exchange. SBHCs improve student attendance and increase access to physical, dental, and mental healthcare [5, 9, 10]. These benefits could be considered in SBHC quality measurements and school accountability metrics to monitor outcomes and guide continuous improvement.

Equity‐focused policies need to go beyond facilitating collaboration and seek to mitigate systemic barriers to care. Our interviewees recognize that economically disadvantaged students in rural communities face greater healthcare needs. While collective action and collective impact frameworks support collaborative networks, they fall short in explicitly addressing equity and social justice concerns. It is important to adopt measures that prioritize underserved communities for SBHC expansion. Regular communication and trust‐building between schools, healthcare systems, and community members are essential to maintaining the collaboration and ensuring that SBHCs are developed in school districts where students need them most.

### 5.1 Limitations

This study has several limitations. First, it is a case study focused on a four‐county rural region in a single state, which may limit the generalizability of the results to other settings and other states with different policies and laws. Second, the study primarily examines the perspectives of schools and a healthcare system, without including opinions from children, families, and other community organizations. Future research could address these limitations by conducting comparative studies with a broader range of SBHCs and gathering perspectives from diverse stakeholders to provide a more comprehensive understanding of the school‐healthcare collaboration in rural settings.

## 6 Conclusions

This study used semi‐structured interviews and thematic analysis to explore how schools and healthcare systems implement and sustain SBHCs across a four‐county rural region in New York State. We found that the strong collaborative culture and shared resources between schools and healthcare systems are essential to establishing and sustaining SBHCs in rural communities. Trust and open communication between healthcare systems and schools facilitate the common goal: to do what is best for students. While SBHCs have the potential to improve access to care and school attendance, challenges around confidentiality, shared measurement, and different priorities persist. Regular meetings, clear boundaries, and a shared goal to enhance student well‐being help sustain school‐healthcare partnerships and SBHCs.

## Ethics Statement

This study was approved and declared exempt by the Cornell Institution Review Board IRB0148254.

## Conflicts of Interest

The authors declare no conflicts of interest.

## Data Availability

The interview data are stored securely in accordance with Institutional Review Board (IRB) approval and are not publicly available to protect participant confidentiality.
